# Clinicopathologic and Genomic Landscape of Non-Small Cell Lung Cancer Brain Metastases

**DOI:** 10.1093/oncolo/oyac094

**Published:** 2022-05-22

**Authors:** Richard S P Huang, Lukas Harries, Brennan Decker, Matthew C Hiemenz, Karthikeyan Murugesan, James Creeden, Khaled Tolba, Laura P Stabile, Shakti H Ramkissoon, Timothy F Burns, Jeffrey S Ross

**Affiliations:** Foundation Medicine, Inc., Cambridge, MA, USA; Foundation Medicine, Inc., Cambridge, MA, USA; Foundation Medicine, Inc., Cambridge, MA, USA; Foundation Medicine, Inc., Cambridge, MA, USA; Foundation Medicine, Inc., Cambridge, MA, USA; Foundation Medicine, Inc., Cambridge, MA, USA; Foundation Medicine, Inc., Cambridge, MA, USA; University of Pittsburgh Medical Center (UPMC) Hillman Cancer Center, University of Pittsburgh, Pittsburgh, PA, USA; Department of Pharmacology & Chemical Biology, University of Pittsburgh, Pittsburgh, PA, USA; Foundation Medicine, Inc., Cambridge, MA, USA; Wake Forest Comprehensive Cancer Center, and Department of Pathology, Wake Forest School of Medicine, Winston-Salem, NC, USA; University of Pittsburgh Medical Center (UPMC) Hillman Cancer Center, University of Pittsburgh, Pittsburgh, PA, USA; Foundation Medicine, Inc., Cambridge, MA, USA; Department of Pathology, State University of New York (SUNY) Upstate Medical University, Syracuse, NY, USA

**Keywords:** non-small cell lung cancer, brain metastasis, genomic

## Abstract

**Background:**

In patients with non-small cell lung cancer (NSCLC), 10%-40% will eventually develop brain metastases. We present the clinicopathologic, genomic, and biomarker landscape of a large cohort of NSCLC brain metastases (NSCLC-BM) samples.

**Materials and Methods:**

We retrospectively analyzed 3035 NSCLC-BM tested with comprehensive genomic profiling (CGP) during routine clinical care. In addition, we compared the NSCLC-BM to a separate cohort of 7277 primary NSCLC (pNSCLC) specimens. Finally, we present data on 67 paired patients with NSCLC-BM and pNSCLC.

**Results:**

Comprehensive genomic profiling analysis of the 3035 NSCLC-BMs found that the most frequent genomic alterations (GAs) were in the *TP53*, *KRAS*, *CDKN2A*, *STK11*, *CDKN2B*, *EGFR*, *NKX2-1*, *RB1*, *MYC*, and *KEAP1* genes. In the NSCLC-BM cohort, there were significantly higher rates of several targetable GAs compared with pNSCLC, including *ALK* fusions, *KRAS* G12C mutations, and *MET* amplifications; and decreased frequency of *MET* exon14 skipping mutations (all *P* < .05). In the subset of NSCLC-BM (*n* = 1063) where concurrent PD-L1 immunohistochemistry (IHC) was performed, 54.7% of the patients with NSCLC-BM were eligible for pembrolizumab based on PD-L1 IHC (TPS ≥ 1), and 56.9% were eligible for pembrolizumab based on TMB-High status. In addition, in a series 67 paired pNSCLC and NSCLC-BM samples, 85.1% (57/67) had at least one additional GA discovered in the NSCLC-BM sample when compared with the pNSCLC sample.

**Conclusions:**

Herein, we defined the clinicopathologic, genomic, and biomarker landscape of a large cohort of patients with NSCLC-BM which can help inform study design of future clinical studies for patients with NSCLC with BM. In certain clinical situations, metastatic NSCLC brain tissue or cerebral spinal fluid specimens may be needed to fully optimize personalized treatment.

## Teaser

Some patients with non-small cell lung cancer (NSCLC) develop brain metastasis. This article examines the genomic landscape of NSCLC brain metastases samples with comprehensive genomic profiling in a large cohort of patients to define the potential applicability of therapeutic advances in NSCLC.

**Implications for Practice**

This report defines the clinicopathologic, genomic, and biomarker landscape of a large cohort of patients with NSCLC-BM, which can help inform study design of future clinical studies for NSCLC patients with BM. In certain clinical situations, metastatic NSCLC brain tissue or cerebral spinal fluid specimens may be needed to fully optimize personalized treatment.

## Introduction

Non-small cell lung cancer (NSCLC) remains a leading cause of morbidity and mortality globally. Currently, 5%-10% of patients with NSCLC have concurrent brain metastases (BM) at time of diagnosis, and 20%-40% will eventually develop BM.^1^ Importantly, multiple studies have shown that the prevalence of *ALK* fusions and *EGFR* mutations is high in patients who eventually develop BM, potentially associated with the extended survival (induced by the advent of the ALK and EGFR inhibitors for patients with NSCLC) allowing for spread of disease to the brain.^2-4^ In addition, central nervous system (CNS) only progression was common in *ALK*-driven NSCLC given the poor CNS penetration of early generation ALK inhibitors.^5^ Finally, the brain is often the primary site of progression, even in patients in which the systemic disease is controlled by targeted therapies or immunotherapy.^5-7^

Multiple targeted therapies for genomic alterations (GAs) in tyrosine kinase-associated genes such as *EGFR*, *ALK*, *ROS1*, *MET*, *RET*, *KRAS*, *BRAF*, and *NTRK1-3* with associated companion diagnostics (CDx) have been developed and are now approved by the United States Food and Drug Administration (FDA) to treat NSCLC.^8^ In addition, testing for multiple emerging targetable biomarkers using next-generation sequencing is currently recommended by the National Comprehensive Cancer Network (NCCN) Guidelines for patients with NSCLC, including *ERBB2* mutations and *MET* amplifications.^9^ Furthermore, multiple immune checkpoint inhibitors (ICPI) have been approved by the FDA for patients with NSCLC with accompanying CDx including pembrolizumab (in patients with tumor mutational burden-high (TMB-High), microsatellite instability-high (MSI-H), or PD-L1 tumor cell expression), atezolizumab (in patients with PD-L1 tumor or immune cell expression), and ipilimumab in combination with nivolumab (in patients with PD-L1 tumor cell expression).^10-14^ Finally, emerging genomic biomarkers for ICPI treatment decision include positive predictive biomarkers like *CD274* amplification and APOBEC mutational signature and negative predictive biomarkers such as *STK11*/*KEAP1* mutations and *MDM2*/*MDM4* amplifications.^15-19^

The preponderance of the initial clinical trials for targeted and immunotherapies in NSCLC were performed on patients without evidence of BM.^1^ However, more recent trials have examined patients with NSCLC-BM with promising findings. For example, *EGFR* and *ALK/ROS1* inhibitors show promising results in patients with NSCLC-BM.^20-24^ Given this, the poor prognosis of NSCLC-BM, and the numerous approved therapies for metastatic NSCLC, the presence of the associated biomarkers for these approved agents within NSCLC-BM warrants further study. We examined the genomic landscape of NSCLC-BM samples with comprehensive genomic profiling (CGP) in a large cohort of patients to define the potential applicability of therapeutic advances in NSCLC.

## Material and Methods

### Patient Cohort

This study was approved by the Western Institutional Review Board Protocol No. 20152817. We performed a retrospective CGP analysis of 3035 consecutive NSCLC-BM samples between August 2014 and June 2020 and compared the results with 7277 unpaired pNSCLC specimens. In addition, the CGP findings in 67 paired NSCLC-BM and pNSCLC samples in the same patient were separately studied. Age, sex, and site of specimen were extracted from accompanying pathology reports.

### Comprehensive Genomic Profiling of Lung Carcinoma Samples

Comprehensive genomic profiling was performed using a hybrid capture-based CGP assay (FoundationOne/FoundationOne CDx) in a Clinical Laboratory Improvement Amendments (CLIA)-certified and College of American Pathologists (CAP)-accredited laboratory (Foundation Medicine, Cambridge, MA) using previously described methods.^25^ FoundationOne/FoundationOne CDx uses a next generation sequencing platform and a hybrid capture methodology that detects base substitutions, insertions/deletions, copy number alterations, and gene rearrangements in up to 324 genes, as well as tumor mutational burden (TMB) and microsatellite instability (MSI). Hematoxylin and eosin (H&E) stained slides from each sample was reviewed by a board-certified pathologist for the presence of adequate tumor (≥20% of nucleated cells are tumor cells) before sequencing. Amplification was defined as ≥ploidy + 4 (copy number 6 for diploid patients) for all genes except for *ERBB2* which was defined as ≥ploidy + 3. TMB was determined on up to 1.14 Mb of sequenced DNA and TMB ≥10 mutations/Mb was considered TMB-High per CDx approval.^12,26^ Microsatellite instability analysis was performed from DNA sequencing up to 114 loci and MSI-High was considered positive.^27,28^ Smoking status was not known for the patients; however, tobacco as well as APOBEC mutational signatures were called as described by Zehir et al^29^ Genomic ancestry of patients was determined using a principle component analysis of genomic single-nucleotide polymorphisms trained on data from the 1000 Genomes Project and each patient was classified as belonging to one of the following super populations: African, Central and South American, East Asian, European, and South Asian.^30,31^

### PD-L1 22C3 Immunohistochemistry Testing

For a subset of cases tested with CGP, PD-L1 immunohistochemistry (IHC) was concurrently performed using the DAKO 22C3 CDx assay per manufacturer’s instructions in a CLIA-certified and CAP-accredited reference laboratory (Foundation Medicine, Morrisville, NC).^32^ Here, DAKO’s tumor proportion scoring (TPS) method was used, where TPS =# PD-L1-positive tumor cells/(total # of PD-L1 positive + PD-L1-negative tumor cells).^33^ We explored both the TPS ≥1 and TPS ≥50 cutoff in this analysis.

### Statistical Analysis

Clinicopathologic differences between different NSCLC cohorts were analyzed using analysis of variance (ANOVA) or Fisher’s exact test. To examine the differences in the genomic landscape of the different cohorts, we identified the top 15 genes that have GAs and compared these same genes with a Fisher’s exact test. In addition, targetable GAs and emerging biomarkers were also compared. *P*-values were adjusted for multiple comparisons using the Bonferroni method and adjusted *P*-values of < 0.05 were considered significant.^34^

## Results

### Clinicopathologic Characteristics of NSCLC-BM

A total of 3035 NSCLC-BM samples were included in this study. Female sex (54.6%. 1656/3025) was significantly more common in NSCLC-BM when compared with pNSCLC (51.0%, 3710/7277) (Fisher’s exact test, *P* = .001). The mean patient age was significantly higher in pNSCLC compared with NSCLC-BM cohort patients (68.5 vs 62.5, respectively, *P* = 1.1E−156; Table 1). East Asian ancestry was more common in the pNSCLC cohort (4.6%, 333/7277) versus the NSCLC-BM cohort (3.4%, 103/3035) (*P* = .031), while no significant difference between the two cohorts was found for other ancestry groups (Table 1). Also, a higher prevalence of adenocarcinoma (69.5% [2110/3035] vs 60.4% [4396/7277]) and large cell neuroendocrine carcinoma (2.6% [79/3,035] vs 1.1% [79/7,277]) and lower prevalence of squamous cell carcinoma (6.9% [209/3035] vs 25.5% [1853/7277]) was found in the NSCLC-BM when compared with pNSCLC (all *P* < .001). Last, there were higher frequencies of the tobacco mutational signature in the NSCLC-BM cohort (20.2% [614/3035] vs 9.8% [715/7277], *P* = 1.0E−04).

**Table 1.** Clinicopathologic characteristic of NSCLC brain metastases (NSCLC-BM) and primary NSCLC (pNSCLC)

Clinicopathologic characteristics	NSCLC-BM (*n* = 3035)	*n*	pNSCLC (*n* = 7277)	*n*	*P*-value
Sex^*^					.001
Male	45.4%	1379	49.0%	3567	
Female	54.6%	1656	51.0%	3710	
Age^**^					1.1E−156
Median	62		69		
Mean	62.5		68.5		
Genetic ancestry^*^					
African	11.1%	337	9.5%	693	.079
Central and South American	5.5%	168	5.3%	382	1
East Asian	3.4%	103	4.6%	333	.031
European	79.5%	2412	79.9%	5811	1
South Asian	0.5%	15	0.8%	58	.605
Histologic subtype^*^					
Adenocarcinoma	69.5%	2110	60.4%	4396	1.0E−17
Adenosquamous carcinoma	0.6%	17	0.9%	67	.568
Carcinosarcoma	0.0%	1	0.1%	8	1
Large cell carcinoma	0.5%	15	0.2%	15	.2
Large cell neuroendocrine carcinoma	2.6%	79	1.1%	79	3.9E−07
NOS	19.3%	586	11.2%	818	2.7E−25
Sarcomatoid carcinoma	0.6%	18	0.6%	41	1
Squamous cell carcinoma	6.9%	209	25.5%	1853	3.0E−118
Mutational Signature^*^					
Tobacco signature	20.2%	614	9.8%	715	1.0E−43

Fisher’s exact test (*P*-values of genetic ancestry and histologic subtype adjusted with Bonferroni method);

ANOVA.

Sixty-seven cases with matched pNSCLC and NCSLC-BM samples were identified. Demographic characteristics were similar for pNSCLC cases with versus without paired samples except for age, which was significantly lower in the paired pNSCLC with paired samples group (60.3 vs 68.2, respectively, *P* = 1.4E−10) (Supplementary Table S1).

### Genomic Landscape of Non-Small Cell Lung Cancer Brain Metastases

**Figure 1.**
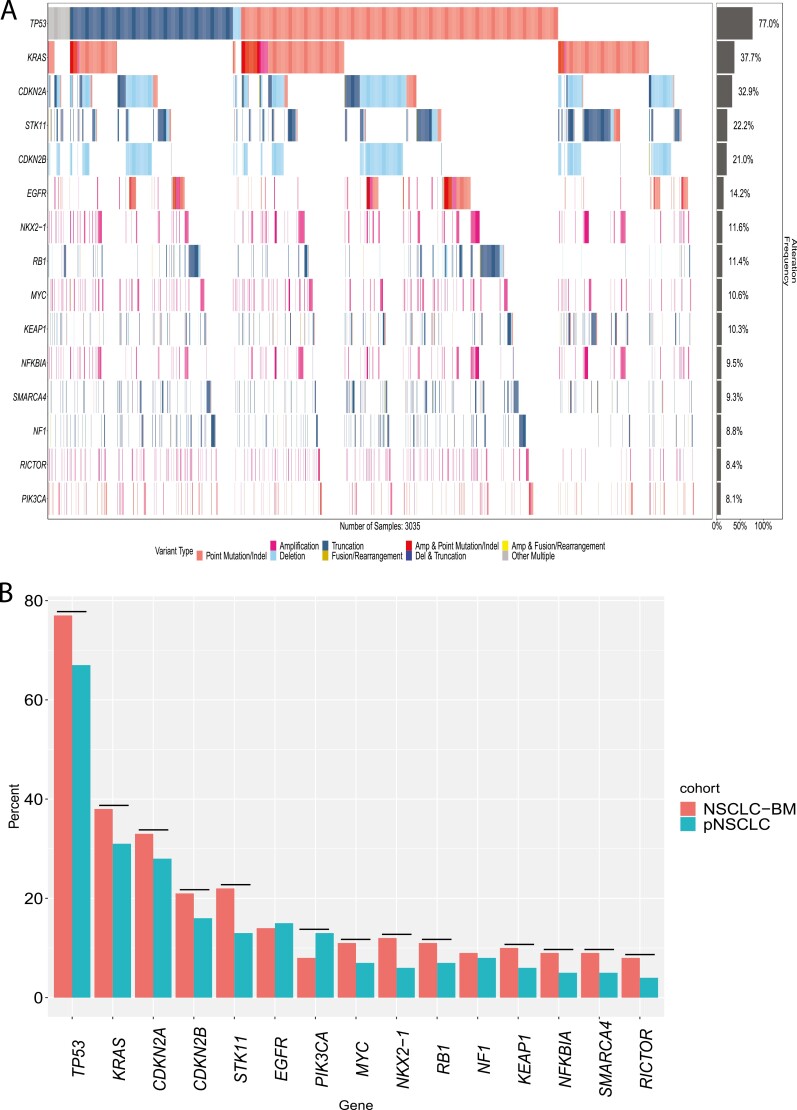
Genomic landscape of non-small cell lung cancer brain metastases (NSCLC-BM). (**A**) Co-mutation plot of the top 15 genes with genomic alterations (GAs) in NSCLC-BM. In the NSCLC-BM cohort (*n* = 3035), the top 15 most altered genes were *TP53* (77.0%, 2337), *KRAS* (37.7%, 1143), *CDKN2A* (32.9%, 998), *STK11* (22.2%, 675), *CDKN2B* (21.0%, 637), *EGFR* (14.2%, 432), *NKX2-1* (11.6%, 353), *RB1* (11.4%, 347), *MYC* (10.6%, 323), *KEAP1* (10.3%, 313), *NFKBIA* (9.5%, 288), *SMARCA4* (9.3%, 283), *NF1* (8.8%, 268), *RICTOR* (8.4%, 256), and *PIK3CA* (8.1%, 245). (B) Longtail plot of the top 15 genes with GAs between the NSCLC-BM and primary NSCLC (pNSCLC). NSCLC-BMs that were significantly enriched for GAs when compared to pNSCLC in *TP53*, *KRAS*, *CDKN2A*, *STK11*, *CDKN2B*, *NKX2-1*, *RB1*, *MYC*, *KEAP1*, *NFKBIA*, *SMARCA4*, *RICTOR*, and decreased for prevalence of *PIK3CA.* Significant differences are designated by horizontal black lines above each comparison group.

Comprehensive genomic profiling analysis of 3035 NSCLC-BM revealed that the 15 most frequently altered genes were: *TP53* (77.0%, 2,337), *KRAS* (37.7%, 1,143), *CDKN2A* (32.9%, 998), *STK11* (22.2%, 675), *CDKN2B* (21.0%, 637), *EGFR* (14.2%, 432), *NKX2-1* (11.6%, 353), *RB1* (11.4%, 347), *MYC* (10.6%, 323), *KEAP1* (10.3%, 313), *NFKBIA* (9.5%, 288), *SMARCA4* (9.3%, 283), *NF1* (8.8%, 268), *RICTOR* (8.4%, 256), and *PIK3CA* (8.1%, 245) (Fig. 1A).

NSCLC-BM (*n* = 3035) was enriched for GAs when compared with pNSCLC (*n* = 7277) in *TP53* (77.0% [2337] vs 66.6% [4845]), *KRAS* (37.7% [1143] vs 31.2% [2271]), *CDKN2A* (32.9% [998] vs 27.7% [2014]), *STK11* (22.2% [675] vs 13.4% [977]), *CDKN2B* (21.0% [637] vs 15.7% [1145]), *NKX2-1* (11.6% [353] vs 6.3% [455]), *RB1* (11.4% [347] vs 7.3% [534]), *MYC* (10.6% [323] vs 6.5% [474]), *KEAP1* (10.3% [313] vs 5.8% [422]) *NFKBIA* (9.5% [288] vs 5.0% [362/7277]), *SMARCA4* (9.3% [283] vs 5.2% [381]), *RICTOR* (8.4% [256] vs 4.5% [324]), and decreased prevalence of *PIK3CA* (8.1% [245] vs 13.0% [944])(all *P* < .0001) (Fig. 1B, Supplementary Table S2).

### Targetable Biomarker Landscape

**Figure 2.**
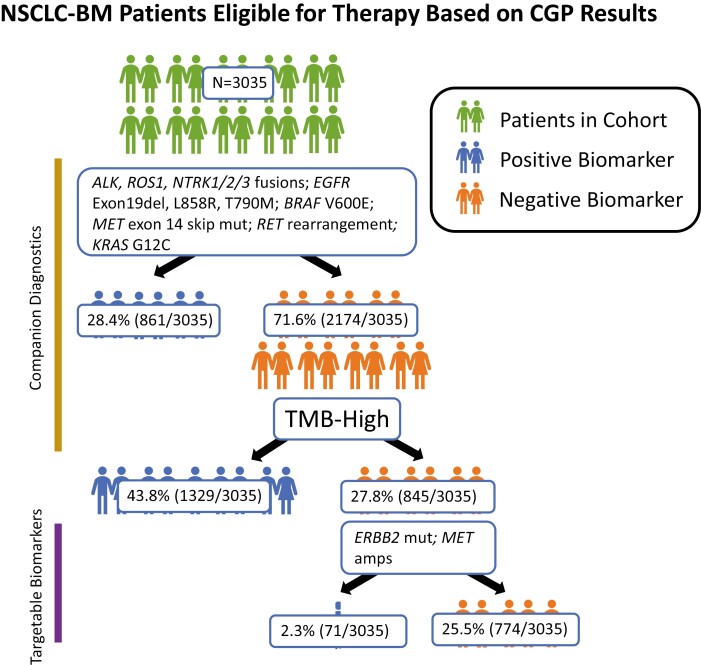
NSCLC brain metastases (NSCLC-BM) patient eligibility for therapy based on biomarker status. Overall, most of the samples (74.5%, 2,261/3,035) in the NSCLC-BM cohort featured one or more CDx-associated biomarker(s) (*ALK*, *ROS1*, *NTRK1/2/3* fusions; *EGFR* Exon19del, L858R, T790M; *BRAF* V600E; *MET* exon 14 skipping mut; *RET* rearrangement; and *KRAS* G12C) or an NCCN recommended targetable biomarker (*ERBB2* mutations; *MET* amplifications). For the 3035 NSCLC-BM patients, 28.4% (861/3035) revealed at least one companion diagnostic-associated GA. Of the remaining 2174 patients, 61.1% (*n* = 1,329) were TMB-High. Last, of the patients without positivity in at least 1 companion diagnostics biomarker, 8.4% (71/845) had positivity in at least one of the NCCN recommended biomarkers.

Overall, most of the samples (74.5%, 2261/3035) in the NSCLC-BM cohort featured one or more CDx-associated biomarker(s) (*ALK*, *ROS1*, *NTRK1/2/3* fusions; *EGFR* Exon19del, L858R, T790M; *BRAF* V600E; *MET* exon 14 skipping mut; *RET* rearrangement; and *KRAS* G12C) or an NCCN recommended targetable biomarker (*ERBB2* mutations; *MET* amplifications) (Fig. 2). For the 3035 patients with NSCLC-BM, 28.4% (861/3035) revealed at least one CDx-associated GA. Of the remaining 2174 patients, 61.1% (*n* = 1329) were TMB-High. Last, of the patients without positivity in at least 1 companion diagnostics biomarker, 8.4% (71/845) had positivity in at least one of the NCCN recommended biomarkers.

In the NSCLC-BM cohort, significantly higher frequencies of several targetable GAs were present when compared with pNSCLC, including *ALK* fusions (2.7% [83/3,035] vs 1.7% [123/7,277]), *KRAS* G12C mutations (15.2% [460/3,035] vs 11.7% [853/7,277]), and *MET* amplifications (4.4% [133/3,035] vs 2.3% [170/7,277]) (all *P* < .05; Table 2). On the other hand, we saw higher rates of *MET* exon14 skipping mutations in pNSCLC compared to the NSCLC-BM (2.3% [169/7277] vs 1.0% [31/3035], *P* = 1.0E−04). The frequencies of the remaining targetable biomarkers were not significantly different between the NSCLC-BM and pNSCLC (*P* ≥ 0.05; Table 2).

**Table 2.** Targetable biomarkers of NSCLC brain metastases (NSCLC-BM) and primary NSCLC (pNSCLC).

Biomarkers	NSCLC-BM (*n* = 3035)	*n*	pNSCLC (*n* = 7277)	*n*	*P*-value
*ALK* fusions	2.7%	83	1.7%	123	.020
*ROS1* fusions	0.3%	10	0.5%	34	1
*EGFR* mutations	10.9%	331	12.8%	929	.191
Exon19del	4.5%	137	5.5%	399	1
L858R	3.2%	97	4.2%	305	.435
T790M	0.6%	18	0.9%	69	1
*BRAF* mutations	4.3%	131	4.4%	318	1
V600E	0.9%	26	1.3%	92	1
*NTRK* fusions	0.1%	4	0.1%	6	1
*MET*ex14 skipping mutations	1.0%	31	2.3%	169	1.1E−04
*RET* rearrangements	0.5%	16	0.7%	48	1
*KRAS* mutations	35.7%	1082	29.6%	2153	4.3E−08
G12C	15.2%	460	11.7%	853	5.9E−05
*ERBB2* mutations	1.9%	59	1.5%	107	1
*MET* amplifications	4.4%	133	2.3%	170	1.4E−06
*ICPI biomarkers*					
TMB-High	55.4%	1680	33.6%	2442	1.3E−91
MSI-H	0.7%	22	0.3%	22	.100
*CD274* amplifications	1.5%	47	1.1%	81	1
*STK11* mutations	17.7%	538	11.6%	847	1.5E−14
*KEAP1* mutations	8.9%	271	5.3%	388	8.5E−10
*MDM2* amplifications	3.3%	100	4.5%	326	.128
*MDM4* amplifications	0.8%	24	0.4%	30	.540
APOBEC mutational signature	6.0%	182	3.9%	286	1.7E−04

Fisher’s exact test (p values adjusted with Bonferroni method).

Last, due to the higher frequencies of adenocarcinoma histologic subtype in the NSCLC-BM cohort compared with the pNSCLC cohort, we examined the targetable biomarkers in the NSCLC-BM with adenocarcinoma histology and compared it with the pNSCLC with adenocarcinoma histology. Here, the trends in the overall NSCLC-BM and pNSCLC comparisons were the same when we specifically examined the adenocarcinoma histology. The targetable biomarkers that were significantly enriched and decreased in the overall NSCLC-BM cohort were also enriched and decreased in the NSCLC-BM adenocarcinoma only cohort, although significance was not reached for all the comparisons (Supplementary Table S3). In addition, while not significantly decreased in the overall NSCLC-BM cohort, *EGFR* exon19 deletions, and *EGFR* L858R mutations were significantly decreased in the NSCLC-BM with adenocarcinoma histology when compared to the pNSCLC with adenocarcinoma histology (both *P* = .001).

### ICPI Biomarkers

Multiple CGP ICPI biomarkers were present in the NSCLC-BM cohort: TMB-High (55.40%, 1680/3035); MSI-H (0.70%, 22/3035), *CD274* amplifications (1.5%, 47/3035); *STK11* mutations (17.70%, 538/3035); *KEAP1* mutations (8.90%, 271/3035); *MDM2* amplifications (3.30%, 100/3035); *MDM4* amplifications (0.80%, 24/3035); and APOBEC mutational signature (6.00%, 182/3035). In addition, ICPI biomarkers of TMB-High (54.4% [1680/3035] vs 33.6% [2442/7277], *P* = 1.30E−91), *STK11* mutations (17.7% [538/3035] vs 11.6% [847/7277], *P* = 1.5E−14), *KEAP1* mutations (8.9% [271/3035] vs 5.3% [388/7277], *P* = 8.5E−10), and APOBEC mutational signature (6.0% [182/3035] vs 3.9% [286/7277], *P* = 1.7E−04) were significantly higher in the NSCLC-BM cohort when compared with the pNSCLC cohort (Table 2).

**Figure 3.**
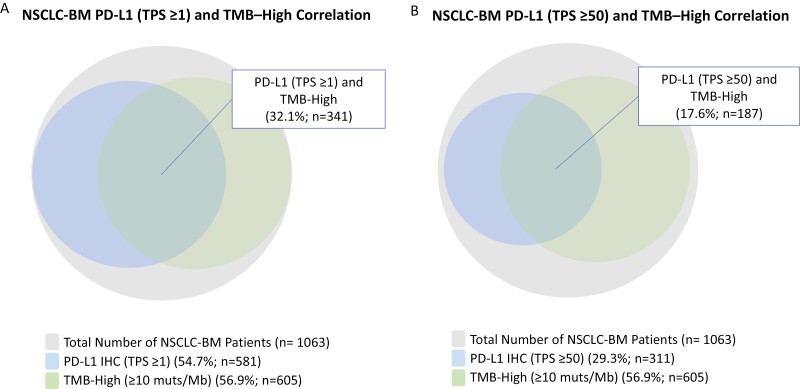
Correlation of PD-L1 tumor cell expression and tumor mutational burden (TMB). (A) When using both comprehensive (CGP) (tumor mutational burden-high [TMB-High]) and PD-L1 immunohistochemistry (IHC) (TPS ≥ 1), 54.7% (581/1063) of the NSCLC-BM patients were eligible for pembrolizumab monotherapy based on PD-L1 IHC, and 56.9% (605/1063) were eligible for pembrolizumab monotherapy based on TMB-High status, and 32.1% (341/1063) of patients positive for both PD-L1 IHC and TMB-High. (B) Using the more stringent PD-L1 IHC scoring cutoff (TPS ≥ 50), 29.3% (311/1,063) of the NSCLC-BM patients were positive PD-L1 IHC, and 17.6% (187/1063) of patients were positive for PD-L1 IHC and TMB-High.

In the subset of NSCLC-BM (*n* = 1063) where concurrent PD-L1 IHC testing was performed, the frequency of PD-L1 expression (TPS ≥ 1) was 54.7% (581/1063) and PD-L1 high expression (TPS ≥ 50) was 29.3% (311/1063) (Fig. 3A,B). When using both CGP (TMB-High) and PD-L1 IHC (TPS ≥ 1), 54.7% (581/1063) of the patients with NSCLC-BM were eligible for pembrolizumab monotherapy based on PD-L1 IHC, and 56.9% (605/1063) were eligible for pembrolizumab monotherapy based on TMB-High status, and 32.1% (341/1063) of patients positive for both PD-L1 IHC and TMB-High (Fig. 3A). Using the more stringent PD-L1 IHC scoring cutoff (TPS ≥ 50), 29.3% (311/1063) of the patients with NSCLC-BM were positive PD-L1 IHC, and 17.6% (187/1063) of patients were positive for PD-L1 IHC and TMB-High (Fig. 3B).

### Paired Primary NSCLC and NSCLC-BM Samples

In our cohort, 67 paired pNSCLC and NSCLC-BM samples were identified (Supplementary Table 4). The time between the sample collection date of the pNSCLC sample to the NSCLC-BM sample ranged from 2 days to more than 5 years (median = 440 days). In aggregate, the NSCLC-BM samples had an additional 106 amplifications, 39 truncations, 33 deletions, 29 short variant mutations, 4 splice site variants, 1 duplication, and 1 fusion detected compared with the paired pNSCLC samples. In addition, of the 67 paired cases, 85.1% (57/67) had at least one additional GA in the NSCLC-BM sample when compared with the paired pNSCLC sample. Also, 61.2% (41/67) of the NSCLC-BM samples did not have at least one GA that was found on the paired pNSCLC sample (Supplementary Table 4).

### Primary NSCLC With and Without Paired Samples

**Figure 4.**
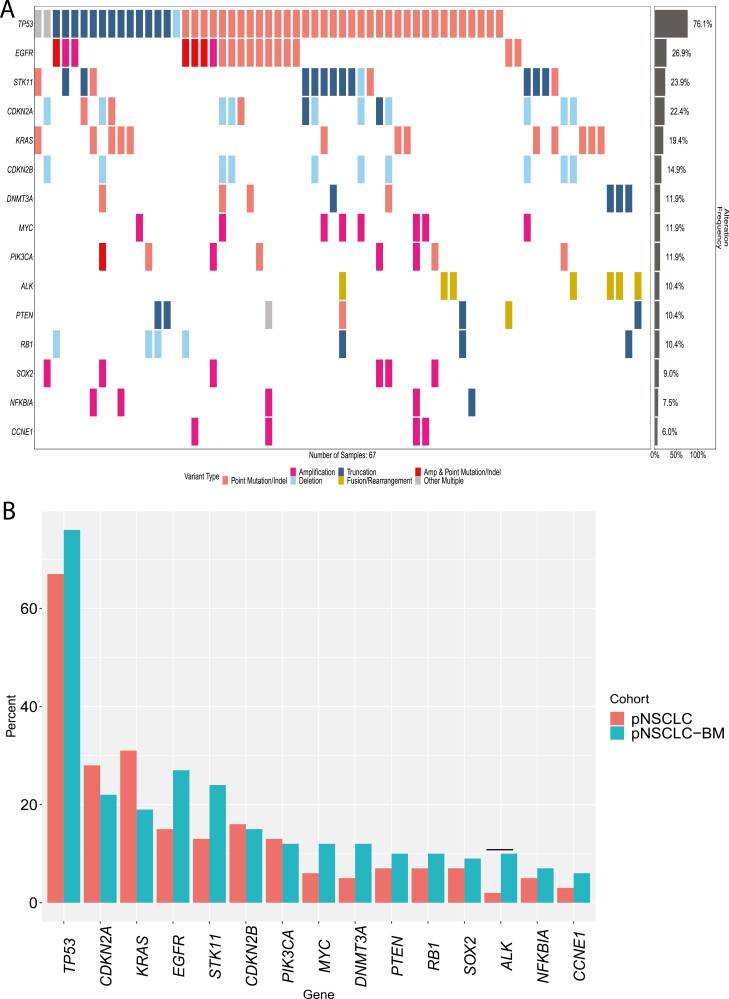
Genomic landscape of 67 primary NSCLC (pNSCLC) with paired brain metastases (BM) samples (that eventually developed brain metastases). (**A**) Co-mutation plot of the top 15 genes with genomic alterations (GAs) in the 67 pNSCLC with paired BM samples (that eventually developed brain metastases). (**B**) Longtail plot of the of the top 15 genes with GAs between the 67 pNSCLC cases with paired BM samples and the pNSCLC without paired BM samples. In the pNSCLC with paired BM samples, we saw a significantly higher prevalence of GAs in *ALK*. pNSCLC-BM are the pNSCLC with paired BM samples and pNSCLC are the samples without paired BM samples. Significant differences are designated by horizontal black lines above each comparison group.

Last, we compared the genomic profiles of the 67 pNSCLC cases with paired BM samples (that eventually metastasized to the brain) with the pNSCLC without paired BM samples (Fig. 4A, B). Here, we saw a significantly higher prevalence of GAs in *ALK* (10.4% [7/67] vs 2.1% [153/7264], *P* = .009), and specifically *ALK* fusions (9.0% [6/67] vs 1.7% [123/7264], *P* = .035), in the pNSCLC with paired BM samples (Fig. 4B, Supplementary Tables S5 and S6).

## Discussion

This retrospective study of 3035 NSCLC-BM revealed the clinicopathologic, genomic, and biomarker profile of NSCLC-BM in a large cohort of patients. Clinicopathologically, the NSCLC-BM cohort was different when compared with the pNSCLC cohort: namely, they were more often younger, females, smokers, or adenocarcinoma or large cell neuroendocrine carcinoma histology. Compared with pNSCLC, NSCLC-BM cases were enriched for alterations in *TP53*, *KRAS*, *CDKN2A*, *STK11*, *CDKN2B*, *NKX2-1, RB1*, *MYC, KEAP1, NFKBIA*, *SMARCA4*, *RICTOR,* and showed decreased prevalence of *PIK3CA* GA. The finding of decreased *PIK3CA* GA in the NSCLC-BM cohort was noteworthy. While *PIK3CA* mutations are currently targetable in breast cancer, there are no anti-*PIK3CA* targeted therapies that are FDA approved for NSCLC.^35^ This finding also suggests a potential rationale for in vitro and clinical investigations into whether *PIK3CA* mutations hinder NSCLC to metastasize to the brain. In sum, these results can be used for future clinical trial design for patients with NSCLC-BM.

Importantly, a significant proportion of the patients (74.5%, 2261/3035) in the NSCLC-BM cohort featured GAs in at least one CDx biomarker or an NCCN recommended targetable biomarker. In the NSCLC-BM cohort, enrichment of *ALK* fusions, *KRAS* G12C mutations, and *MET* amplifications; and decrease of *MET* exon14 skipping mutations were identified. Given that the full clinical history of the patients was not available, it is not possible to confirm whether the enrichment of *ALK* fusions, *KRAS* G12C, and *MET* amplification in the NSCLC-BM samples is due to longer survival of the patients having been treated with systemic therapy targeting these GAs. Moreover, it is not likely that the *KRAS* G12C mutations and *MET* amplifications are associated with a propensity of these patients to develop BM associated with longer survival for these patients in that there are no FDA approvals for these GAs during the time frame of this study. Also, while *MET* exon14 skipping mutations were less frequent in the NSCLC-BM cohort, 31 (1.0%) patients in the NSCLC-BM cohort still harbored this potentially targetable alteration. In sum, these data suggest that if clinically feasible, CGP could be performed from a brain tissue or cerebral spinal fluid (CSF) specimen in patients with NSCLC-BM to identify targetable alterations in the BM. Of note, while the CGP results of blood liquid biopsy has shown considerable concordance with tissue biopsies and has shown clinical utility in many metastatic diseases, several studies have demonstrated that the blood-brain barrier (BBB) may limit the quantity of ctDNA that is shed into the circulating blood plasma, rendering blood liquid biopsy limited in its ability to detect the genomic profile of the NSCLC-BM.^36^

In the 67-paired primary pNSCLC and NSCLC-BM samples, we found that there was at least one additional GA in 85.1% (57/67) NSCLC-BM (post-treatment) sample when compared with the paired pNSCLC; and 61.2% (41/67) of the NSCLC-BM lost at a least one GA when compared with the paired pNSCLC sample. This further adds evidence to the different genomic profiles between pNSCLC and NSCLC-BM and illustrates the importance of performing CGP on the NSCLC-BM when clinically feasible. Last, we observed a significantly higher prevalence of *ALK* fusions in the pNSCLC with a paired NSCLC-BM (that eventually metastasized to the brain) when compared with pNSCLC without a paired NSCLC-BM. This is consistent with the literature that patients with *ALK* fusions have a predilection of developing BM, likely due to the longer survival of patients on ALK inhibitors and having time to develop BM.^2,3^

The ICPI biomarker landscape in NSCLC is complex with multiple CDx indications as well as exploratory biomarkers. While the ICPI landscape in NSCLC and other tumor types was explored in numerous other studies, the literature is more lacking in NSCLC-BM.^11,37-39^ In the NSCLC-BM samples, enrichment of ICPI-positive predictive biomarkers of TMB-High and APOBEC mutational signature were found. However, enrichment of ICPI-negative predictive biomarkers of *STK11* and *KEAP1* were also discovered, exemplifying the importance of testing with CGP to identify both positive and negative predictive biomarkers for ICPI in these patients. Of note, the observed finding of higher TMB, *STK11*, and *KEAP1* might represent overall adverse prognostic markers and predictive of more aggressive disease, rather than a finding specific to brain metastases. For a subset of patients with NSCLC-BM, we also had concurrent PD-L1 IHC (*n* = 1063) results. Here, we identified similar prevalence rates of PD-L1 tumor cell expression as previously reported.^40^ Of interest, we discovered that when using both CGP and PD-L1 IHC, these CDx biomarkers identified independent subsets of patients that are potentially eligible for pembrolizumab, with some overlap suggesting that it is important to test with both PD-L1 IHC and CGP to identify the NSCLC-BM patients that are eligible for pembrolizumab.

The major strength of this study is the large number (>3000) of NSCLC-BM samples, all of which underwent centralized CGP. However, a primary limitation of this study is the incomplete clinical information available for patient samples and the smaller number of cases with paired pNSCLC and NSCLC-BM samples. It is likely that some of the pNSCLC samples in our study could also have concurrent NSCLC-BM; however, in the absence of clinical histories, we do not know the extent of their disease. In addition, since most BMs are not sampled and are treated with radiotherapy, there is likely some sampling bias for NSCLC-BMs in relatively younger patients with good performance status (who are able to undergo a procedure for tissue acquisition) and symptomatic BM as opposed to elderly frail patients with NSCLC-BM whose BM were not symptomatic.

## Conclusion

Herein, we defined the clinicopathologic, genomic, and biomarker landscape of a large cohort of patients with NSCLC-BM which can help inform study design of future clinical studies for patients with NSCLC with BM. In certain clinical situations, metastatic NSCLC brain tissue or cerebral spinal fluid specimens may be needed to fully optimize personalized treatment.

## Supplementary Material

oyac094_suppl_Supplementary_Materials

## Data Availability

The data underlying this article are available in the article and in its online supplementary material.
