# Supplementary material for: Clinicopathologic and Genomic Landscape of Non-Small Cell Lung Cancer Brain Metastases
Source: Oncologist. 2022 May 22;27(10):839–48. doi: 10.1093/oncolo/oyac094 (PMC9526503; doi:10.1093/oncolo/oyac094)
Supplement: oyac094_suppl_Supplementary_Materials [file oyac094_suppl_supplementary_materials.docx]

**Supplement**

**Supplemental Table 1** Clinicopathologic characteristic of primary NSCLC (pNSCLC) with paired brain metastases (BM) samples and pNSCLC without paired BM samples

| **Clinicopathologic Characteristics** | **pNSCLC with paired BM samples (n=67)** | **n** | **pNSCLC without paired BM samples (n=7264)** | **n** | **p-value** |
| --- | --- | --- | --- | --- | --- |
| **Sex*** |  |  |  |  | 0.065 |
| *Male* | 37.3% | 25 | 49.0% | 3561 |  |
| *Female* | 62.7% | 42 | 51.0% | 3703 |  |
| **Age**** |  |  |  |  | 1.4E-10 |
| *Median* | 62 |  | 69 |  |  |
| *Mean* | 60.3 |  | 68.2 |  |  |
| **Genetic Ancestry*** |  |  |  |  |  |
| *African* | 3.0% | 2 | 9.5% | 693 | 0.445 |
| *Central and South American* | 6.0% | 4 | 5.2% | 381 | 1 |
| *East Asian* | 10.4% | 7 | 4.6% | 332 | 0.171 |
| *European* | 80.60% | 54 | 79.80% | 5800 | 1 |
| *South Asian* | 0.0% | 0 | 0.8% | 58 | 1 |
| **Histologic subtype*** |  |  |  |  |  |
| *Adenocarcinoma* | 64.2% | 43 | 60.4% | 4384 | 1 |
| *Adenosquamous carcinoma* | 1.5% | 1 | 0.9% | 67 | 1 |
| *Carcinosarcoma* | 0.0% | 0 | 0.1% | 8 | 1 |
| *Large cell carcinoma* | 0.0% | 0 | 0.2% | 15 | 1 |
| *Large cell neuroendocrine carcinoma* | 1.5% | 1 | 1.1% | 79 | 1 |
| *NOS* | 13.4% | 9 | 11.3% | 818 | 1 |
| *Sarcomatoid carcinoma* | 0.0% | 0 | 0.6% | 41 | 1 |
| *Squamous cell carcinoma* | 19.4% | 13 | 25.5% | 1852 | 1 |
| **Mutational Signature*** |  |  |  |  |  |
| *Tobacco signature* | 16.4% | 11 | 9.8% | 714 | 0.095 |

*Fisher's exact test p values of genetic ancestry and histologic subtype adjusted with Bonferroni method; **ANOVA

**Supplement Table 2.** Prevalence of top 15 genes with genomic alterations between the NSCLC brain metastases (NSCLC-BM) and primary NSCLC (pNSCLC) cohort

| **Genes** | **NSCLC-BM (n=3035)** | **n** | **pNSCLC (n= 7277)** | **n** | ***p-value** |
| --- | --- | --- | --- | --- | --- |
| *TP53* | 77.0% | 2337 | 66.6% | 4845 | 2.7E-25 |
| *KRAS* | 37.7% | 1143 | 31.2% | 2271 | 4.6E-09 |
| *CDKN2A* | 32.9% | 998 | 27.7% | 2014 | 2.2E-06 |
| *STK11* | 22.2% | 675 | 13.4% | 977 | 3.3E-26 |
| *CDKN2B* | 21.0% | 637 | 15.7% | 1145 | 3.8E-09 |
| *EGFR* | 14.2% | 432 | 15.4% | 1123 | 1 |
| *NKX2-1* | 11.6% | 353 | 6.3% | 455 | 4.9E-18 |
| *RB1* | 11.4% | 347 | 7.3% | 534 | 6.3E-10 |
| *MYC* | 10.6% | 323 | 6.5% | 474 | 5.4E-11 |
| *KEAP1* | 10.3% | 313 | 5.8% | 422 | 5.5E-14 |
| *NFKBIA* | 9.5% | 288 | 5.0% | 362 | 1.6E-15 |
| *SMARCA4* | 9.3% | 283 | 5.2% | 381 | 1.4E-12 |
| *NF1* | 8.8% | 268 | 7.7% | 557 | 0.697 |
| *RICTOR* | 8.4% | 256 | 4.5% | 324 | 1.8E-13 |
| *PIK3CA* | 8.1% | 245 | 13.0% | 944 | 4.4E-12 |

*Fisher’s Exact Test (p values adjusted with Bonferroni method)

**Supplemental Table 3.** Targetable Biomarkers of NSCLC brain metastases (NSCLC-BM) with adenocarcinoma histology and primary NSCLC (pNSCLC) with adenocarcinoma histology

| **Biomarkers** | **NSCLC-BM Adenocarcinoma Histology (n=2110)** | **n** | **pNSCLC Adenocarcinoma Histology (n=4396)** | **n** | ***p-value** |
| --- | --- | --- | --- | --- | --- |
| ***ALK* fusions** | 3.1% | 65 | 2.5% | 110 | 1 |
| ***ROS1* fusions** | 0.5% | 10 | 0.6% | 26 | 1 |
| ***EGFR* mutations** | 12.7% | 268 | 18.9% | 832 | 4.2E-09 |
| Exon19del | 5.4% | 113 | 8.2% | 361 | 0.001 |
| L858R | 3.9% | 82 | 6.4% | 282 | 0.001 |
| T790M | 0.8% | 17 | 1.5% | 66 | 0.419 |
| ***BRAF* mutations** | 4.7% | 100 | 5.9% | 259 | 1 |
| V600E | 1.1% | 23 | 1.9% | 82 | 0.472 |
| ***NTRK* fusions** | 0.1% | 2 | 0.1% | 4 | 1 |
| ***MET*ex14 skipping mutations** | 0.9% | 20 | 2.5% | 111 | 2.6E-04 |
| ***RET r*earrangements** | 0.7% | 14 | 0.9% | 40 | 1 |
| ***KRAS* mutations** | 42.1% | 888 | 40.2% | 1769 | 1 |
| G12C | 18.3% | 387 | 18.1% | 794 | 1 |
| ***ERBB2* mutations** | 2.0% | 43 | 2.1% | 92 | 1 |
| ***MET* amplifications** | 4.3% | 90 | 2.6% | 116 | 0.015 |
| **ICPI biomarkers** |  |  |  |  |  |
| TMB-High | 54.3% | 1146 | 29.5% | 1298 | 1.4E-80 |
| MSI-H | 0.5% | 10 | 0.1% | 6 | 0.339 |
| *CD274* amplifications | 1.5% | 31 | 0.6% | 25 | 0.012 |
| *STK11* mutations | 20.7% | 436 | 15.7% | 688 | 1.8E-05 |
| *KEAP1* mutations | 9.4% | 199 | 5.7% | 251 | 1.5E-06 |
| *MDM2* amplifications | 3.5% | 74 | 5.1% | 223 | 0.098 |
| *MDM4* amplifications | 0.8% | 17 | 0.5% | 20 | 1 |
| APOBEC mutational signature | 6.2% | 131 | 3.4% | 148 | 6.4E-06 |

*Fisher’s Exact Test (p values adjusted with Bonferroni method)

**Supplemental Table 4.** Sixty-seven Paired Primary NSCLC (pNSCLC) and NSCLC Brain Metastases (NSCLC-BM)

| **Diagnosis** | **Patient age at primary samples collection** | **Days between specimen collection** | **Genetic Ancestry** | **pNSCLC** | **NSCLC-BM** | **Additional GA** | **GA Lost** |
| --- | --- | --- | --- | --- | --- | --- | --- |
| Lung adeno | 64 | 96 | EUR | *TP53* R175L, *KRAS* G12D, *KDM5C* Q1554*, *PTPRO* M1I | *MYC* amp, *EPHB4* amp, *TP53* R175L, *KRAS* G12D, *KDM5C* Q1554*, *ERRFI1* I158fs*3, *PTPRO* M1I | *MYC* amp, *EPHB4* amp, *ERRI1* I158fs*3 | N/A |
| Lung adeno | 62 | 381 | EUR | *TP53* deletion, *KDM5A* amp, *BRAF* G469A, *MLL-MSANTD2* fusion, *PTCH1-PTCH1* deletion | *C17orf39* amp, *KDM5A* amplification, *TP53* deletion, *BRAF* G469A, *PTCH1-PTCH1* deletion | *C17orf39* amp | *MLL-MSANTD2* fusion |
| Lung adeno | 68 | 633 | AMR | *AURKA* amp, *ARFRP1* amp, *GNAS* amp, *TP53* R158L, *ASXL1* V1367I, *KRAS* G12C, *STK11* G196V, *TP53* splice site 673-1G>T | *MCL1* amp, *GNAS* amp, *ARFRP1* amp, *AURKA* amp, *ZNF217* amp, *NTRK1* amp, *STK11* G196V, *TP53* R158L, *KRAS* G12C, *TP53* splice site 673-1G>T | *MCL1* amp*, ZNF217* amp*, NTRK1* amp | *ASXL1* V1367I |
| Lung adeno | 72 | 444 | EUR | *ERBB2* amp, *NKX2-1* amp, *CDKN2B* deletion, *CDKN2A* deletion, *RAD21* amp, *MYC* amp, *FGFR3-TACC3* fusion, *TP53* E285K, *EGFR* T790M, *EGFR* L747_A755>SKG, *DNMT3A* S714C | *NKX2-1* amp, *ERBB2* amp, *EGFR* L747_A755>SKG, *TP53* E285K | N/A | *CDKN2B* deletion*, CDKN2A* deletion*, RAD21* amp*, MYC* amp*, FGFR3-TACC3* fusion*, EGFR* T790M*, DNMT3A* S714C |
| Lung adeno | 77 | 257 | EAS | *TP53* R158L, *STK11* E165*, *SDHA* A331T, *MRE11A-PIWIL4* truncation, *NF1* Q535*, *KDM5C* S1112fs*31 | *NFKBIA* amp, *LYN* amp, *SOX2* amp, *MTAP* deletion, *TERC* amp, *CDKN2A* deletion, *NKX2-1* amp, *PRKCI* amp, *CDKN2B* deletion, *STK11* E165*, *TP53* R158L, *KDM5C* S1112fs*31, *NF1* Q535* | *NFKBIA* amp, *LYN* amp, *SOX2* amp, *MTAP* deletion, *TERC* amp, *CDKN2A* deletion, *NKX2-1* amp, *PRKCI* amp, *CDKN2B* deletion | *SDHA* A331T, *MRE11A-PIWIL4* truncation |
| Lung adeno | 51 | 176 | EUR | *MET* amp, *EGFR* E746_A750del, *EGFR* T790M, *TP53* S241F, *CDKN2A* H83Y, *MET* amp, *CDKN2A* H83Y, *EGFR* E746_A750del, *TP53* S241F | *CDK6* amp, *MET* amp, *CDKN2A* H83Y, *EGFR* E746_A750del, *TP53* S241F, *PIK3CA* Q75E | *CDK6* amp*, PIK3CA* Q75E | *EGFR* E746_A750del, *EGFR* T790M |
| Lung adeno | 74 | 31 | EUR | *CDKN2A* deletion, *CDKN2B* deletion, *ERBB4* P1025S, *TP53* P152L, *NPM1* splice site 583-1G>T, *STK11* C158fs*4 | *CDKN2B* deletion, *CDKN2A* deletion, *NF2* deletion, *ERBB4* P1025S, *TP53* P152L, *NPM1* splice site 583-1G>T, *STK11* C158fs*4 | *NF2* deletion | *N/A* |
| Lung adeno | 76 | 456 | EUR | *CCNE1* amp, *MYC* amp, *NFKBIA* amp, *PIK3CA* amp, *RAD21* amp, *KEL* R248W, *TERC* amp, *PRKCI* amp, *LYN* amp, *TP53* P278R | *PIK3CB* amp, *SOX2* amp, *SMARCA4-N/A* truncation, *CCNE1* amp, *PRKCI* amp, *MYC* amp, *LYN* amp, *TERC* amp, *TP53* P278R | *PIK3CB* amp, *SOX2* amp, *SMARCA4-N/A* truncation | *NFKBIA* amp, *PIK3CA* amp, *RAD21* amp, *KEL* R248W |
| Lung adeno | 63 | 1269 | EUR | *KRAS* G12C, *KEAP1* G417R | *ERBB4* amp, *RAD21* amp, *MYC* amp, *AKT3* amp, *KEAP1* G417R, *KRAS* G12C | *ERBB4* amp, *RAD21* amp, *MYC* amp, *AKT3* amp | *N/A* |
| Lung adeno | 59 | 419 | EUR | *TP53* R280I, *TET2* K1090fs*16 | *NKX2-1* amp, *GATA6* amp, *KEAP1* deletion, *CRKL* amp, *CTNNB1-ULK4* truncation, *SMARCA4* Q418_E419>H*, *BCORL1* P366fs*52, *TP53* R280I, *TET2* K1090fs*16 | *NKX2-1* amp, *GATA6* amp, *KEAP1* deletion, *CRKL* amp, *CTNNB1-ULK4* truncation, *SMARCA4* Q418_E419>H*, *BCORL1* P366fs*52 | *N/A* |
| Lung adeno | 65 | 372 | EUR | *CDKN2A* deletion, *MTAP* deletion, *MYC* amp, *CDKN2B* deletion, *STK11* R86* | *CDKN2A* deletion, *MTAP* deletion, *MYC* amp, *CDKN2B* deletion, *STK11* R86* | *N/A* | *N/A* |
| Lung adeno | 64 | 3179 | EUR | *CDKN2A* A20T, *PBRM1* E184D, *ASXL1* Q512*, *STK11* P38fs*13, *TP53* splice site 673-1G>T | *EMSY* amp, *CCNE1* amp, *AKT2* amp, *MCL1* amp, *CRKL* amp, *FGF12* amp, *PIK3CG* amp, *DNMT3A* P904L, *RAD50* E271fs*34, *CDKN2A* A20T, *STK11* P38fs*13, *TP53* splice site 673-1G>T | *EMSY* amp, *CCNE1* amp, *AKT2* amp, *MCL1* amp, *CRKL* amp, *FGF12* amp, *PIK3CG* amp, *DNMT3A* P904L, *RAD50* E271fs*34 | *PBRM1* E184D, *ASXL1* Q512* |
| Lung adeno | 80 | 441 | EUR | *DNMT3A* R771Q, *EGFR* L861Q, *TP53* G245R, *TP53* Y236C, *CTCF* E149fs*5 | *DNMT3A* R771Q, *EGFR* L861Q, *TP53* G245R, *TP53* Y236C, *CTCF* E149fs*5 | *N/A* | *N/A* |
| Lung adeno | 62 | 1469 | EUR | *ZNF217* amp, *ARFRP1* amp, *KRAS* G12C | *STK11* deletion, *ARFRP1* amp, *KRAS* G12C, *SMARCA4-SMARCA4* duplication | *STK11* deletion*, SMARCA4-SMARCA4 duplication* | *ZNF217* amp |
| Lung adeno | 51 | 481 | EUR | *ERBB2* R678Q, *CDKN2A* R80*, *ERBB2* R970W, *MLL3* K2797fs*26, *SMARCB1* R155H, *TSHR* E575K, *TP53* E171K, *TP53* R110L, *MLH1* D450fs*41, *NOTCH4* H1230fs*74, *STK11* splice site 735-2A>G | *ERBB2* R970W, *TP53* E171K, *FOXP1* P83H, *MYC* amp, *GNAS* R160C, *IL7R* R267fs*28, *PDGFRA* V499M, *RNF43* R286W, *TAF1* R243H, *TSC2* R57H, *ATRX* P2448H, *CDK12* R1331*, *ARID1A* D1850fs*33, *ATRX* K1357fs*18, *BARD1* D172fs*40, *CHD4* K1681fs*3, *CHD4* K73fs*129, *EP300* E1320*, *FANCL* splice site 217-1G>T, *TAF1* splice site 2567-1G>T, *TET2* K326fs*21, *TP53* R110L, *CDKN2A* R80*, *ERBB2* R678Q, *MLH1* D450fs*41, *NOTCH4* H1230fs*74, *STK11* splice site 735-2A>G | *FOXP1* P83H, *MYC* amp, *GNAS* R160C, *IL7R* R267fs*28, *PDGFRA* V499M, *RNF43* R286W, *TAF1* R243H, *TSC2* R57H, *ATRX* P2448H, *CDK12* R1331*, *ARID1A* D1850fs*33, *ATRX* K1357fs*18, *BARD1* D172fs*40, *CHD4* K1681fs*3, *CHD4* K73fs*129, *EP300* E1320*, *FANCL* splice site 217-1G>T, *TAF1* splice site 2567-1G>T, *TET2* K326fs*21 | *MLL3* K2797fs*26, *SMARCB1* R155H, *TSHR* E575K |
| Lung adeno | 44 | 393 | EUR | *KRAS* G12D, *MSH2* Q793*, *SETD2* T1483fs*33, *TP53* splice site 97-1G>A | *BRCA2* deletion, *NFKBIA* amp, *NKX2-1* amp, *BRAF* amp, *KEL* amp, *KRAS* G12D, *MSH2* Q793*, *SETD2* T1483fs*33, *TP53* splice site 97-1G>A | *BRCA2* deletion, *NFKBIA* amp, *NKX2-1* amp, *BRAF* amp, *KEL* amp | *N/A* |
| Lung adeno | 46 | 1126 | EAS | *EGFR* L858R, *TP53* R273H | *CDKN2A* deletion, *MET* amp, *NKX2-1* amp, *MTAP* deletion, *CDKN2B* deletion, *TP53* R273H, *EGFR* L858R | *CDKN2A* deletion, *MET* amp, *NKX2-1* amp, *MTAP* deletion, *CDKN2B* deletion | *N/A* |
| Lung adeno | 54 | 885 | AMR | *CCNE1* amp, *EGFR* amp, *TP53* C275F, *EGFR* E746_A750del | *EGFR* amp, *NFKBIA* amp, *NKX2-1* amp, *EGFR* T790M, *CDC73* R126*, *CCNE1* amp, *TP53* C275F, *EGFR* E746_A750del | *NFKBIA* amp, *NKX2-1* amp, *EGFR* T790M, *CDC73* R126* | *N/A* |
| Lung adeno | 38 | 90 | EUR | *ALK-EML4* fusion, *MUTYH* G382D, *TNF* D121N, *DNMT3A* Q100fs*24 | *CDKN2B* deletion, *CDKN2A* deletion, *AKT3* amp, *ALK-EML4* fusion, *MUTYH* G382D, *TNF* D121N, *DNMT3A* Q100fs*24 | *CDKN2B* deletion, *CDKN2A* deletion, *AKT3* amp | *N/A* |
| Lung adeno | 64 | 1846 | EUR | *ALK-MTA3* fusion, *DNMT3A* R143* | *STK11* deletion, *CREBBP* S1761*, *ALK-MTA3* fusion, *DNMT3A* R143* | *STK11* deletion, *CREBBP* S1761* | *N/A* |
| Lung adeno | 60 | 567 | EUR | *NFKBIA* amp, *KIT* amp, *NKX2-1* amp, *TP53* E298*, *KRAS* G12C, *ATM* splice site 1235+1G>A, *TSC2* E399* | *KDR* amp, *PDGFRA* amp, *DNMT3A* S714C, *KRAS* amp, *KIT* amp, *NKX2-1* amp, *NFKBIA* amp, *TP53* E298*, *KRAS* G12C, *TSC2* E399*, *ATM* splice site 1235+1G>A | *KDR* amp, *PDGFRA* amp, *DNMT3A* S714C | *N/A* |
| Lung adeno | 48 | 335 | EAS | *RB1* deletion, *EGFR* amp, *EGFR* L747_P753>S, *RBM10* S781fs*8, *TP53* Y107fs*16 | *EGFR* amp, *EGFR* L747_P753>S, *RB1* deletion, *RBM10* S781fs*8, *TAF1* S111*, *TP53* Y107fs*16 | *TAF1* S111* | *N/A* |
| Lung adeno | 66 | 894 | AMR | *ZNF217* amp, *ARFRP1* amp, *EGFR* E746_A750del, *PIK3CA* N1044K, *TP53* E285K, *TP53* R267W, *TP53* I232T | *MYC* amp, *RAD21* amp, *ZNF217* amp, *ARFRP1* amp, *PIK3CA* N1044K, *TP53* I232T, *EGFR* E746_A750del | *MYC* amp, *RAD21* amp | *TP53* E285K, *TP53* R267W |
| Lung adeno | 44 | 10 | EUR | *IGF2* amp, *HRAS* amp, *NF2* L163fs*40, *ALK-EML4* fusion, *TP53* S241F, *U2AF1* S34F | *CDKN2A* deletion, *CDKN2B* deletion, *ALK-EML4* fusion, *TP53* S241F, *U2AF1* S34F | *CDKN2A* deletion, *CDKN2B* deletion | *IGF2* amp, *HRAS* amp, *NF2* L163fs*40 |
| Lung adeno | 78 | 1590 | EUR | *TP53* V157F, *KRAS* G12C, *CUL3* splice site 1842+1G>T | *CBL* E366K, *KRAS* G12C, *TP53* V157F, *CUL3* splice site 1842+1G>T | *CBL* E366K | *N/A* |
| Lung adeno | 52 | 2 | EUR | *KIT* amp, *KDR* amp, *EGFR* amp, *FLT1* E487K, *TP53* A69fs*56 | *FGF19* amp, *CCND1* amp, *FGF4* amp, *FGF3* amp, *MCL1* amp, *ZNF217* amp, *PDGFRA* amp, *ERRFI1* D165fs*10, *KIT* amp, *KDR* amp, *EGFR* amp, *TP53* A69fs*56 | *FGF19* amp, *CCND1* amp, *FGF4* amp, *FGF3* amp, *MCL1* amp, *ZNF217* amp, *PDGFRA* amp, *ERRFI1* D165fs*10 | *FLT1* E487K |
| Lung adeno | 42 | 780 | EAS | *CCNE1* amp, *CDK6* amp, *MYC* amp, *TP53* R175H | *CCNE1* amp, *TP53* R175H | *N/A* | *CDK6* amp, *MYC* amp |
| Lung adeno | 73 | 956 | EUR | *FRS2* amp, *APC* D849fs*12, *ACVR1B* R225fs*6, *MDM2* amp, *EGFR* E746_A750del, *PTEN*-*PTEN* deletion, *FUBP1* splice site 837+1delG | *MDM2* amp, *AXIN1* G514R, *APC* E1309fs*4, *EGFR* E746_A750del, *PTEN*-*PTEN* deletion, *FUBP1* splice site 837+1delG | *AXIN1* G514R, *APC* E1309fs*4 | *FRS2* amp, *APC* D849fs*12, *ACVR1B* R225fs*6 |
| Lung adeno | 70 | 399 | EUR | *MYC* amp, *AXIN1* W85*, *KIT* G102C, *KRAS* G12V, *STK11* Q220*, *TP53* R249T, *GATA1* E200* | *CDKN2B* deletion, *CDKN2A* deletion, *KIT* G102C, *STK11* Q220*, *KRAS* G12V, *AXIN1* W85*, *TP53* R249T, *GATA1* E200* | *CDKN2B* deletion, *CDKN2A* deletion | *MYC* amp |
| Lung adeno | 64 | 408 | AFR | *RICTOR* amp, *FGF10* amp, *CDKN2A* deletion, *CDKN2B* deletion, *ALK-EML4* fusion, *MUTYH* Y165C | *CDKN2A* deletion, *NKX2-1* amp, *MYC* amp, *TP53* T211fs*36, *STK11-N/A* truncation, *CDKN2B* deletion, *ALK-EML4* fusion, *MUTYH* Y165C | *NKX2-1* amp, *MYC* amp, *TP53* T211fs*36, *STK11-N/A* truncation | *RICTOR* amp, *FGF10* amp |
| Lung adeno | 69 | 682 | EUR | *EGFR* L747_A750>P, *TP53* A276D, *SMAD4* S178* | *FGF19* amp, *FGF3* amp, *ARFRP1* amp, *FGF4* amp, *EGFR* amp, *MYCN* amp, *CCND1* amp. *AKT2* amp, *RB1* deletion, *TP53* A276D, *EGFR* L747_A750>P | *FGF19* amp, *FGF3* amp, *ARFRP1* amp, *FGF4* amp, *EGFR* amp, *MYCN* amp, *CCND1* amp. *AKT2* amp, *RB1* deletion | *SMAD4* S178* |
| Lung adeno | 72 | 652 | EUR | *STK11* D194Y, *KRAS* G12V, *ATRX* Q2406*, *SMARCA4* D1299fs*5 | *CCND1* amp, *FGF19* amp, *ERBB4* amp, *FGF4* amp, *FGF3* amp, *KRAS* G12V, *STK11* D194Y, *SMARCA4* D1299fs*5, *ATRX* Q2406* | *CCND1* amp, *FGF19* amp, *ERBB4* amp, *FGF4* amp, *FGF3* amp | *N/A* |
| Lung adeno | 67 | 701 | EUR | *STK11* G242V, *TP53* V157F, *BRAF* G469A, *FANCA* splice site 1827-1G>A | *CDKN2A* deletion, *CDKN2B* deletion, *MTAP* deletion, *DNMT3A* R635W, *ARID1A* Q581*, *BRAF* G469A, *STK11* G242V, *TP53* V157F, *FANCA* splice site 1827-1G>A | *CDKN2A* deletion, *CDKN2B* deletion, *MTAP* deletion, *DNMT3A* R635W, *ARID1A* Q581* | *N/A* |
| Lung adeno | 58 | 1094 | EUR | *CDKN2B* deletion, *CDKN2A* deletion, *SOX2* amp, *CYLD* S371*, *TP53* splice site 375G>T, *FBXW7* R367*, *TP53* H179L, *NF1-NF1* deletion | *AURKB* amp, *C17orf39* amp, *NFKBIA* amp, *CCNE1* amp, *FGF10* amp, *NKX2-1* amp, *RICTOR* amp, *TP53* R273L, *BCORL1* M644fs*4 | *AURKB* amp, *C17orf39* amp, *NFKBIA* amp, *CCNE1* amp, *FGF10* amp, *NKX2-1* amp, *RICTOR* amp, *TP53* R273L, *BCORL1* M644fs*4 | *CDKN2B* deletion, *CDKN2A* deletion, *SOX2* amp, *CYLD* S371*, *TP53* splice site 375G>T, *FBXW7* R367*, *TP53* H179L, *NF1-NF1* deletion |
| Lung adeno | 62 | 190 | EAS | *CDK4* amp, *GLI1* amp, *PDGFRA* amp, *FRS2* amp, *MDM2* amp, *EGFR* E746_A750del, *MLL2-N/A* truncation | *CDKN2A* deletion, *CDKN2B* deletion, *MET* amp, *RET-KIF5B* fusion, *TP53* M237I, *MLL2* C1534Y | *CDKN2A* deletion, *CDKN2B* deletion, *MET* amp, *RET-KIF5B* fusion, *TP53* M237I, *MLL2* C1534Y | *CDK4* amp, *GLI1* amp, *PDGFRA* amp, *FRS2* amp, *MDM2* amp, *EGFR* E746_A750del, *MLL2-N/A* truncation |
| Lung adeno | 44 | 445 | EAS | *CDKN2A* deletion, *EMSY* amp, *RICTOR* amp, *FGF10* amp, *CDKN2B* deletion, *MET* amp, *TP53* G245S, *EGFR* L858R | *CDKN2A* deletion, *CDKN2B* deletion, *MET* amp, *EMSY* amp, *MTAP* deletion, *EGFR* amp, *RICTOR* amp, *FGF10* amp, *TP53* G245S, *EGFR* L858R | *MTAP* deletion, *EGFR* amp | *N/A* |
| Lung adeno | 59 | 498 | EUR | *TP53* R158L, *CYLD-CMTM4* truncation, *TAF1* Q1109* | *RICTOR* amp, *FGF10* amp, *AR* amp, *RAD51D-TXNDC11* truncation, *TP53* R158L | *RICTOR* amp, *FGF10* amp, *AR* amp, *RAD51D-TXNDC11* truncation | *CYLD-CMTM4* truncation, *TAF1* Q1109* |
| Lung adeno | 39 | 92 | EUR | *EGFR* amp, *MYST3* amp, *RB1* deletion, *EGFR* E746_A750del, *TP53* V143M, *FANCF* F48fs*42 | *RB1* deletion, *AR* amp, *BRCA2* S1882*, *PIK3CA* E545K, *EGFR* E746_A750del, *TP53* V143M, *FANCF* F48fs*42 | *AR* amp, *BRCA2* S1882*, *PIK3CA* E545K | *EGFR* amp, *MYST3* amp |
| Lung adeno | 81 | 652 | EUR | *TP53* E258Q, *APC* I1307K, *STK11* splice site 465-2A>T, *BLM* Y736fs*5, *DNMT3A* S352fs*55 | *MYC* amp, *DIS3* amp, *MCL1* amp, *MLL2-N/A* truncation, *TP53* E258Q, *APC* I1307K, *STK11* splice site 465-2A>T | *MYC* amp, *DIS3* amp, *MCL1* amp, *MLL2-N/A* truncation | *BLM* Y736fs*5, *DNMT3A* S352fs*55 |
| Lung adeno | 69 | 573 | EUR | *EGFR* amp, *BRAF* G469A, *EGFR* L858R, *TP53* C176F | *EGFR* amp, *HGF* amp, *MTAP* deletion, *CDKN2A* deletion, *CCNE1* amp, *CDKN2B* deletion, *EGFR* L858R, *TP53* C176F | *HGF* amp, *MTAP* deletion, *CDKN2A* deletion, *CCNE1* amp, *CDKN2B* deletion | *BRAF* G469A |
| Lung adeno | 62 | 362 | EUR | *EGFR* amp, *IGF2R* amp, *SPTA1* splice site 2806-1G>T, *TP53* E271*, *NTRK3* P526Q, *STK11* splice site 734+1G>T, *INPP4B* splice site 688+1G>T | *EGFR* amp, *CCNE1* amp, *TP53* E271*, *NTRK3* P526Q, *INPP4B* splice site 688+1G>T, *STK11* splice site 734+1G>T | *CCNE1* amp | *IGF2R* amp, *SPTA1* splice site 2806-1G>T |
| Lung adeno | 68 | 959 | EUR | *KRAS* G12C | *CDKN2A* deletion, *KRAS* G12C | *CDKN2A* deletion | *N/A* |
| Lung adeno | 67 | 368 | EUR | *CTNNB1* S45F, *STK11* E165*, *KRAS* G12D, *EZH2* K270*, *ATM* N1140fs*11 | *CTNNB1* S45F, *KRAS* G12D, *STK11* E165*, *ARID1A* Y560fs*55, *ATM* N1140fs*11, *EZH2* K270* | *ARID1A* Y560fs*55 | *N/A* |
| Lung adeno | 46 | 354 | EUR | *STK11* deletion, *KIT* amp, *EPHA3* amp, *LRP1B* splice site 5209+1G>T, *CDKN2B* deletion, *CDKN2A* deletion, *MYC* amp, *TP53* N239S, *TP53* H193R, *KEAP1* splice site 1708+1G>T, *NF1* splice site 7062+1G>T | *CDKN2A* deletion, *RAD21* amp, *MTAP* deletion, *CDKN2B* deletion, *STK11* deletion, *MYC* amp, *TP53* N239S, *TP53* H193R, *NF1* splice site 7062+1G>T, *KEAP1* splice site 1708+1G>T | *RAD21* amp, *MTAP* deletion | *KIT* amp, *EPHA3* amp, *LRP1B* splice site 5209+1G>T |
| Lung large cell neuroendocrine carcinoma | 48 | 324 | EAS | *NFKBIA* amp, *NKX2-1* amp, *CCNE1* amp, *EGFR* L858R, *PTEN* C136Y, *PTEN* Y336*, *TP53* A138P | *NKX2-1* amp, *CCNE1* amp, *NFKBIA* amp, *TP53* A138P, *PTEN* C136Y, *EGFR* L858R | *N/A* | *PTEN* Y336* |
| Lung large cell neuroendocrine carcinoma | 77 | 7 | EUR | *RB1* deletion, *PTEN* G165*, *BRCA2* S1982fs*22, *NOTCH2* P6fs*27, *MAP3K1* E1152*, *KDM6A* splice site 443+2T>G, *TP53* splice site 376-2A>G | *RB1* deletion, *RICTOR* amp, *HGF* amp, *FGF10* amp, *DICER1-N/A* truncation, *NOTCH2* P6fs*27, *PTEN* G165*, *KDM6A* splice site 443+2T>G, *MAP3K1* E1152*, *TP53* splice site 376-2A>G | *RICTOR* amp, *HGF* amp, *FGF10* amp, *DICER1-N/A* truncation | *BRCA2* S1982fs*22 |
| NSCLC (NOS) | 60 | 255 | EUR | *PARK2* splice site 735-1_735GG>TT, *STK11* splice site 598-1G>T | *CDKN2A* deletion, *KRAS* G13C | *CDKN2A* deletion, *KRAS* G13C | *PARK2* splice site 735-1_735GG>TT, *STK11* splice site 598-1G>T |
| NSCLC (NOS) | 66 | 1939 | EUR | *TP53* E224*, *KRAS* G12V, *CDKN2A* D108Y, *PARK2* N52fs*29 | *PARK2* N52fs*29 | N/A | *TP53* E224*, *KRAS* G12V, *CDKN2A* D108Y |
| NSCLC (NOS) | 51 | 311 | AMR | *ALK-KIF5B* fusion, *PTEN* splice site 164+1G>T | *HGF* amp, *ZNF703* amp, *WHSC1L1* amp, *TP53* Y234_S241del, *ALK-KIF5B* fusion | *HGF* amp, *ZNF703* amp, *WHSC1L1* amp, *TP53* Y234_S241del | *PTEN* splice site 164+1G>T |
| NSCLC (NOS) | 67 | 327 | EUR | *NFKBIA* amp, *MYCL1* amp, *NKX2-1* amp, *JAK2* amp, *CD274* amp, *PDCD1LG2* amp, *KRAS* G13C, *STK11* G242W, *NCOR1* Q345*, *RUNX1* E143*, *TP53* splice site 920-2A>T | *NKX2-1* amp, *NFKBIA* amp, *MYCL1* amp, *STK11* G242W, *KRAS* G13C, *NCOR1* Q345*, *RUNX1* E143*, *TP53* splice site 920-2A>T | *N/A* | *JAK2* amp, *CD274* amp, *PDCD1LG2* amp |
| NSCLC (NOS) | 55 | 1163 | EUR | *ALK-EML4* fusion, *TP53* V173M, *SETD2* S299fs*2 | *FRS2* amp, *MDM2* amp, *MITF* amp, *FOXP1* amp, *GRIN2A* R1022H, *CEBPA*-N/A truncation, *KEAP1* T400fs*14, *ALK-EML4* fusion, *TP53* V173M, *SETD2* S299fs*2 | *FRS2* amp, *MDM2* amp, *MITF* amp, *FOXP1* amp, *GRIN2A* R1022H, *CEBPA*-N/A truncation, *KEAP1* T400fs*14 | *N/A* |
| NSCLC (NOS) | 45 | 440 | EUR | *FGF10* amp, *RICTOR* amp, *SMAD2* R427*, *TRRAP* A2419V, *EP300* D1399N, *GLI1* R113Q, *RB1* R358*, *SMAD4* R497H, *TP53* R273H, *CREBBP* R1173*, *PTEN* R130* | *SMAD4* R497H, *CREBBP* R1173*, *TP53* R273H, *PTEN* R130*, *RB1* R358* | *N/A* | *FGF10* amp, *RICTOR* amp, *SMAD2* R427*, *TRRAP* A2419V, *EP300* D1399N, *GLI1* R113Q |
| NSCLC (NOS) | 46 | 301 | EUR | *MYC* amp, *PTEN* H93Q, *TP53* C238F, *ALK-N/A* rearrangement, *RB1* splice site 2521-2A>G, *STK11* Q214* | *ARFRP1* amp, *IGF1R* amp, *MYC* amp, *PTEN* H93Q, *TP53* C238F, *ALK-N/A* rearrangement, *STK11* Q214*, *RB1* splice site 2521-2A>G | *ARFRP1* amp, *IGF1R* amp | *N/A* |
| NSCLC (NOS) | 74 | 241 | EUR | *MYC* amp, *CCND1* T286I, *TP53* K139fs*31, *MLL3* M3416_Q3417>I*, *SMAD2* splice site 1135+1G>T, *SMARCA4* E685*, *ARID1A* M1634fs*1 | *ZNF217* amp, *PTPN11* G503V, *APC* Q1338*, *TP53* G266V, *CDKN2A* S43fs*9 | *ZNF217* amp, *PTPN11* G503V, *APC* Q1338*, *TP53* G266V, *CDKN2A* S43fs*9 | *MYC* amp, *CCND1* T286I, *TP53* K139fs*31, *MLL3* M3416_Q3417>I*, *SMAD2* splice site 1135+1G>T, *SMARCA4* E685*, *ARID1A* M1634fs*1 |
| NSCLC (NOS) | 64 | 451 | EUR | *PRKDC* S702F, *PTEN* T319fs*24, *TP53* Q144fs*20, *RAD51C* splice site 838-1G>C | *RICTOR* amp, *STK11* deletion, *FGF10* amp, *PTEN* T319fs*24, *TP53* Q144fs*20, *RAD51C* splice site 838-1G>C | *RICTOR* amp, *STK11* deletion, *FGF10* amp | *PRKDC* S702F |
| NSCLC (NOS) | 75 | 510 | EUR | *TP53* R213G, *EGFR* K745_E746insVPVAIK | *TP53* R213G, *EGFR* K745_E746insVPVAIK | *N/A* | *N/A* |
| NSCLC (NOS) | 49 | 382 | EUR | *CDK4* amp, *GLI1* amp, *TOP2A* amp, *PTPRD* P265fs*3, *ERBB2* amp, *ERBB3* amp, *PTPN11* G503V, *FUBP1* splice site 1184-2A>T, *DNMT3A* E482*, *KEAP1* G379fs*36, *RB1* splice site 940-1G>A | *ERBB2* amp, *ERBB3* amp, *CDK4* amp, *PTPN11* G503V, *STAG2-N4BP2L1* truncation, *DNMT3A* E482*, *FUBP1* splice site 1184-2A>T, *KEAP1* G379fs*36, *RB1* splice site 940-1G>A | *STAG2-N4BP2L1* truncation | *GLI1* amp, *TOP2A* amp, *PTPRD* P265fs*3 |
| Lung SCC | 68 | 275 | EUR | *PIK3CA* amp, *CDKN2A* deletion, *SOX2* amp, *FGF12* amp, *CDKN2B* deletion, *FGF14* amp, *DNMT3A* R882C, *U2AF1* Q157R, *TP53* P301fs*44, *NFE2L2* G31A, *PIK3CA* E542K, *MLL2* Q3973* | *CDKN2A* deletion, *CDKN2B* deletion, *DNMT3A* R882C, *U2AF1* Q157R, *NFE2L2* G31A, *TP53* P301fs*44, *PIK3CA* E542K, *MLL2* Q3973* | N/A | *PIK3CA* amp, *SOX2* amp, *FGF12* amp, *FGF14* amp |
| Lung SCC | 60 | 427 | EUR | *CDKN2B* deletion, *MTAP* deletion, *CDK6* amp, *CDKN2A* deletion, *NF2* S87*, *RICTOR* E542D, *PIK3CA* E545K, *DAXX* E207*, *NOTCH1* S1511* | *PTEN* deletion, *CDKN2B* deletion, *CDK6* amp, *CDKN2A* deletion, *RET* amp, *MTAP* deletion, *PIK3CA* E542K, *NF2* splice site 1575-1G>C | *PTEN* deletion*, RET* amp, *NF2* splice site 1575-1G>C | *NF2* S87**, RICTOR* E542D, *DAXX* E207*, *NOTCH1* S1511* |
| Lung SCC | 59 | 427 | EUR | *PRKCI* amp, *TERC* amp, *FGFR1* amp, *CDKN2A* deletion, *SOX2* amp, *CDKN2B* deletion, *ZNF703* amp, *WHSC1L1* amp, *DNMT3A* R882H, *TP53* R175H | *CDKN2A* deletion, *SOX2* amp, *WHSC1L1* amp, *PRKCI* amp, *FGFR1* amp, *CDKN2B* deletion, *REL* amp, *TP53* K132E, *PDGFRB* V823I, *ZNF703* amp, *TERC* amp, *TP53* R175H, *DNMT3A* R882H | *REL* amp, *TP53* K132E, *PDGFRB* V823I | *N/A* |
| Lung SCC | 65 | 357 | EUR | *SOX2* amp, *TP53* V172D, *PIK3CA* E545K | *SOX2* amp, *TP53* V172D, *PIK3CA* E545K, *TP53* Y205D, *NFE2L2* R34Q, *SOCS1* P36fs*75 | *TP53* Y205D, *NFE2L2* R34Q, *SOCS1* P36fs*75 | *N/A* |
| Lung SCC | 50 | 939 | EUR | *ZNF703* amp, *WHSC1L1* amp, *BCL2L2* amp, *FGFR1* amp, *RB1* deletion, *PIK3CA* G1049R, *MAPK1* E322K, *NFE2L2* D29N, *TP53* P89fs*34 | *ZNF703* amp, *RB1* deletion, *FGFR1* amp, *WHSC1L1* amp, *BCL2L2* amp, *MAPK1* E322K, *PIK3CA* G1049R, *NFE2L2* D29N, *TP53* P89fs*34, *MLL2* E5312* | *MLL2* E5312* | *N/A* |
| Lung SCC | 42 | 533 | EUR | *CREBBP* R1173*, *CDKN1A* R143W, *ARID1A* Q775*, *CTNNA1* L825fs*49 | *CDKN1A* R143W, *NCOR1* S177*, *PIK3CA* E545K, *ARID2* S1302*, *CTNNA1* S441* , *EP300* splice site 3329_3501+307del480 | *NCOR1* S177*, *PIK3CA* E545K, *ARID2* S1302*, *CTNNA1* S441* , *EP300* splice site 3329_3501+307del480 | *CREBBP* R1173**, ARID1A Q775*, CTNNA1 L825fs*49* |
| Lung SCC | 64 | 672 | EUR | *PIK3CA* amp, *SOX2* amp. *FGF19* amp, *FGF4* amp, *FGF3* amp, *NOTCH2* P6fs*27, *TP53* V274F, *CCND1* amp, *CDKN2A* Y44fs*1, *HSD3B1* G40R, *NFE2L2* R34Q, *CCND1* amp, *TP53* V274F, *ACVR1B* S323fs*2 | *FGFR4* amp, *CCND1* amp, *CDKN2A* Y44fs*1, *TP53* V274F, *NFE2L2* R34Q, *HSD3B1* G40R, *CCND1* amp, *ACVR1B* S323fs*2 | *FGFR4* amp | *PIK3CA* amp, *SOX2* amp. *FGF19* amp, *FGF4* amp, *FGF3* amp, *NOTCH2* P6fs*27, *TP53* V274F |
| Lung SCC | 49 | 234 | AFR | *FGFR1* amp, *ZNF703* amp, *RAF1* amp, *PDGFRA* E1032D, *TP53* R273L, *NF2* E186*, *SMARCA4* splice site 3952-1G>T, *WT1* C303*, *LRP1B* splice site 7762_7762+1GG>AT | *MYCN* amp, *KDM6A* deletion, *ZNF703* amp, *FGFR1* amp, *PDGFRA* E1032D, *TP53* R273L, *LRP1B* splice site 7762_7762+1GG>AT, *NF2* E186*, *SMARCA4* splice site 3952-1G>T, *WT1* C303* | *MYCN* amp, *KDM6A* deletion | *RAF1* amp |
| Lung SCC | 57 | 445 | EUR | *SOX2* amp, *CDK6* amp, *HGF* amp, *PRKCI* amp, *TERC* amp, *EGFR* amp, *BRCA2-BRCA2* duplication, *FGF12* amp, *PIK3CA* amp, *TP53* R273C | *FGF12* amp, *PIK3CA* amp, *SOX2* amp, *TP53* R273C, *CHEK2* I157T, *NFE2L2* R34Q | *CHEK2* I157T, *NFE2L2* R34Q | *CDK6* amp, *HGF* amp, *PRKCI* amp, *TERC* amp, *EGFR* amp, *BRCA2-BRCA2* duplication |
| Lung SCC | 53 | 3 | EUR | *TP53* R273H, *NF2* R198*, *NFKBIA* W66* | *MYCN* amp, *TP53* R273H, *NF2* R198*, *NFKBIA* W66* | *MYCN* amp | *N/A* |

**Supplemental Table 5.** Prevalence of top 15 genes with genomic alterations between the primary NSCLC (pNSCLC) with paired brain metastases (BM) samples and pNSCLC without paired BM samples

| **Gene** | **pNSCLC with paired BM samples (n=67)** | **n** | **pNSCLC without paired BM samples (n=7264)** | **n** | ***p-value** |
| --- | --- | --- | --- | --- | --- |
| *TP53* | 76.1% | 51 | 66.6% | 4837 | 1 |
| *EGFR* | 26.9% | 18 | 15.4% | 1119 | 0.243 |
| *STK11* | 23.9% | 16 | 13.4% | 972 | 0.275 |
| *CDKN2A* | 22.4% | 15 | 27.7% | 2009 | 1 |
| *KRAS* | 19.4% | 13 | 31.2% | 2269 | 0.686 |
| *CDKN2B* | 14.9% | 10 | 15.7% | 1141 | 1 |
| *DNMT3A* | 11.9% | 8 | 4.8% | 349 | 0.232 |
| *MYC* | 11.9% | 8 | 6.5% | 471 | 1 |
| *PIK3CA* | 11.9% | 8 | 13.0% | 941 | 1 |
| *ALK* | 10.4% | 7 | 2.1% | 153 | 0.009 |
| *PTEN* | 10.4% | 7 | 6.6% | 480 | 1 |
| *RB1* | 10.4% | 7 | 7.4% | 534 | 1 |
| *SOX2* | 9.0% | 6 | 7.0% | 506 | 1 |
| *NFKBIA* | 7.5% | 5 | 5.0% | 361 | 1 |
| *CCNE1* | 6.0% | 4 | 2.7% | 193 | 1 |

*Fisher’s Exact Test (p values adjusted with Bonferroni method)

**Supplemental Table 6.** Targetable Biomarkers of primary NSCLC (pNSCLC) with paired brain metastasis (BM) samples and pNSCLC without paired BM samples

| **Biomarkers** | **pNSCLC with paired BM samples (n=67)** | **n** | **pNSCLC without paired BM samples (n=7264)** | **n** | ***p-value** |
| --- | --- | --- | --- | --- | --- |
| ***ALK* fusions** | 9.0% | 6 | 1.7% | 123 | 0.025 |
| ***ROS1* fusions** | 0.0% | 0 | 0.5% | 34 | 1 |
| ***EGFR* mutations** | 22.4% | 15 | 12.7% | 925 | 0.603 |
| Exon19del | 13.4% | 9 | 5.4% | 395 | 0.246 |
| L858R | 6.0% | 4 | 4.2% | 305 | 1 |
| T790M | 3.0% | 2 | 0.9% | 67 | 1 |
| ***BRAF* mutations** | 4.5% | 3 | 4.4% | 318 | 1 |
| V600E | 0.0% | 0 | 1.3% | 92 | 1 |
| ***NTRK* fusions** | 0.0% | 0 | 0.1% | 6 | 1 |
| ***MET*ex14 skipping mutations** | 0.0% | 0 | 2.3% | 169 | 1 |
| ***RET* rearrangements** | 0.0% | 0 | 0.6% | 44 | 1 |
| ***KRAS* mutations** | 19.4% | 13 | 29.6% | 2151 | 1 |
| G12C | 9.0% | 6 | 11.7% | 852 | 1 |
| ***ERBB2* mutations** | 1.5% | 1 | 1.5% | 107 | 1 |
| ***MET* amplifications** | 3.0% | 2 | 2.3% | 169 | 1 |
| ***ICPI biomarkers*** |  |  |  |  |  |
| TMB-High | 35.8% | 24 | 33.5% | 2437 | 1 |
| MSI-H | 0.0% | 0 | 0.3% | 22 | 1 |
| *CD274* amplificatons | 1.5% | 1 | 1.1% | 80 | 1 |
| *STK11* mutations | 22.4% | 15 | 11.6% | 842 | 0.265 |
| *KEAP1* mutations | 4.5% | 3 | 5.3% | 387 | 1 |
| *MDM2* amplifications | 3.0% | 2 | 4.5% | 326 | 1 |
| *MDM4* amplifications | 0.0% | 0 | 0.4% | 30 | 1 |
| APOBEC mutational signature | 6.0% | 4 | 3.9% | 284 | 1 |

*Fisher’s Exact Test (p values adjusted with Bonferroni method)
